# Sex-specific risks of death in patients hospitalized for hyponatremia: a population-based study

**DOI:** 10.1007/s12020-019-02073-x

**Published:** 2019-09-02

**Authors:** Buster Mannheimer, Jakob Skov, Henrik Falhammar, Jan Calissendorff, Jonatan D. Lindh, David Nathanson

**Affiliations:** 1 Department of Clinical Science and Education at Södersjukhuset, Karolinska Institutet, Stockholm, Sweden; 2 Department of Molecular Medicine and Surgery, Karolinska Institutet, Stockholm, Sweden; 3 Department of Endocrinology, Metabolism and Diabetes, Karolinska University Hospital, Stockholm, Sweden; 4 Department of Laboratory Medicine, Division of Clinical Pharmacology, Karolinska University Hospital Huddinge, Karolinska Institutet, Stockholm, Sweden; 5 Department of Medicine, Karolinska University Hospital Huddinge, Karolinska Institutet, Stockholm, Sweden

**Keywords:** Hyponatremia, SIADH, Mortality, Population-based study, Hospitalization

## Abstract

**Purpose:**

Several studies have reported an association between hyponatremia and lethality. However, it remains elusive whether hyponatremia independently contributes to lethality. The aim of the study was to investigate associations between hyponatremia and lethality and differences in lethality between men and women hospitalized due to hyponatremia.

**Methods:**

Four registries were utilized in this population-based retrospective study: The National Patient Registry, the Cause of Death Register, the Swedish Prescribed Drug Register and the Total Population Register (NPR) from which the controls were sampled. All hospitalized patients with a first-ever principal ICD10 diagnosis of hyponatremia or syndrome of inappropriate ADH secretion in the NPR between 1 October 2005 and 31 December 2014 were defined as cases. Cox regression with adjustment for potential confounders was used.

**Results:**

14,359 individuals with a principal diagnosis of hyponatremia, and 57,382 matched controls were identified. Median age was 76 years and the majority were women (72%). Median age for women and men was 79 and 68 years, respectively. Adjusted hazard ratios (and 95% CI) for lethality in those with hyponatremia compared with controls were for the entire population 5.5 (4.4–7.0) and in the subgroup free from previously known underlying disease 6.7 (3.3–13.3). Lethality in women with hyponatremia was lower compared with men: HR: 0.56 (0.49–0.64). In the healthier group the lethality remained lower for women: HR: 0.49 (0.34–0.71).

**Conclusions:**

Patients hospitalized due to hyponatremia faced an increased subsequent lethality that was independent of concomitant disease. This increase was nearly twice as large among men compared with women.

## Introduction

Hyponatremia is the most frequently encountered electrolyte disorder in hospitalized patients [1] and is usually defined as a serum concentration of ≤135 mEq/L [1, 2]. Changes in sodium concentrations are associated with a number of diseases and have been linked to an increased lethality due to heart failure, liver cirrhosis, kidney failure, and cancer [3–7].

The prevalence of hyponatremia has been shown to be higher in women [8, 9]. However, data on whether gender influences hyponatremia-associated lethality is utterly scarce. Over the years, several studies have investigated the prognosis of hospitalized patients with hyponatremia [10]. Nevertheless, very few, if any, have reported the outcome in a larger population hospitalized *primarily* for hyponatremia. Moreover, it still remains elusive whether hyponatremia independently contributes to lethality, or if hyponatremia merely is a surrogate marker for the severity of an underlying disease [10].

The objective of the present study was to investigate the association between hospitalization due to hyponatremia and subsequent lethality with special emphasis on differences between the genders.

## Material and methods

### Design and setting

The study was a Swedish nation-wide retrospective observational registry study.

### Data sources

This study utilized data from four registries: The National Patient Registry (NPR) covering all diagnoses with ICD codes at hospital admissions and specialist out-patient visits, the Cause of Death Register, the Swedish Prescribed Drug Register covering all drug prescriptions filled, encoded using Anatomical Therapeutic Chemical (ATC) codes and the Total Population Register from which the controls were sampled. Individual patient-level data from the registers were linked and matched using the unique Swedish personal identification number.

### Study population and definitions

All hospitalized patients with a first-ever principal *International Classification of Diseases*, 10th Revision (ICD10) diagnosis of E87.1 (hyponatremia) or E22.2 (syndrome of inappropriate ADH secretion [SIADH]) in the NPR between 1 October 2005 and 31 December 2014 were defined as cases and the date for hospitalization was set as index date. Controls matched for age, sex, and municipality (four controls per case) who had not been diagnosed with hyponatremia since 1 January 1997 were selected from the Total Population Register and were assigned the same index date as their matched case. The study population in the present study has been described in detail previously [9, 11, 12]. In the present study, the study population was followed onward from the index date and was investigated with regard to occurring deaths until 31 December 2014. Comorbidities and treatment, defined by ICD10 diagnoses and ATC codes were identified for cases and controls at the index date. To further investigate whether hyponatremia is independently associated with lethality we selected a population with all cases without any recorded comorbidity and matched controls for age, sex, and municipality (four controls per case).

### Outcome

The outcome was defined as time to death (all-cause lethality) within 1 year after the index date.

### Variables

In the multivariable Cox regression analysis, adjustments were made for age, renal disease, cardiovascular disease, liver disease, adrenal failure, congestive obstructive pulmonary disease (COPD), pneumonia, diabetes, alcoholism, frailty, cancer, and polypharmacy (>3 drugs). ICD10 codes and ATC codes were used to define potential confounders (definitions presented in the appendix). The variables included in the final multivariable models were confounders with a potential influence on both exposure (hyponatremia) and outcome (1-year lethality).

### Statistical analysis

The primary analysis was a survival analysis with a Cox proportional hazards model, using the index date as time point 0 and a follow-up time of 1 year. Both univariable and multivariable cox regression was used to analyze the association between hyponatremia and lethality. In an attempt to further explore whether hyponatremia is independently associated with lethality, subgroup analyses were performed for individuals without recorded comorbidity (i.e., diagnoses other than E87.1 or E22.2). An interaction term between gender and hyponatremia was created to further explore potential gender differences in the association between hyponatremia and lethality. Kaplan–Meier curves were plotted with pooled data to show all-cause lethality. Adjustment for age, renal disease, cardiovascular disease, liver disease, adrenal failure, COPD, pneumonia, diabetes, alcohol abuse, frailty, cancer, use of thiazide diuretics, ACE/ARB, SSRI, antiepileptic drugs, beta blockers, antiarrhythmic agents, and anticoagulants were performed when all individuals were included in the dataset. In subgroup analyses with the individuals without other comorbidity, adjustments were restricted to age, frailty, and polypharmacy. *P*-values <0.05 were considered significant. All calculations were performed using R version 3.3.2.

## Results

**Fig. 1:**
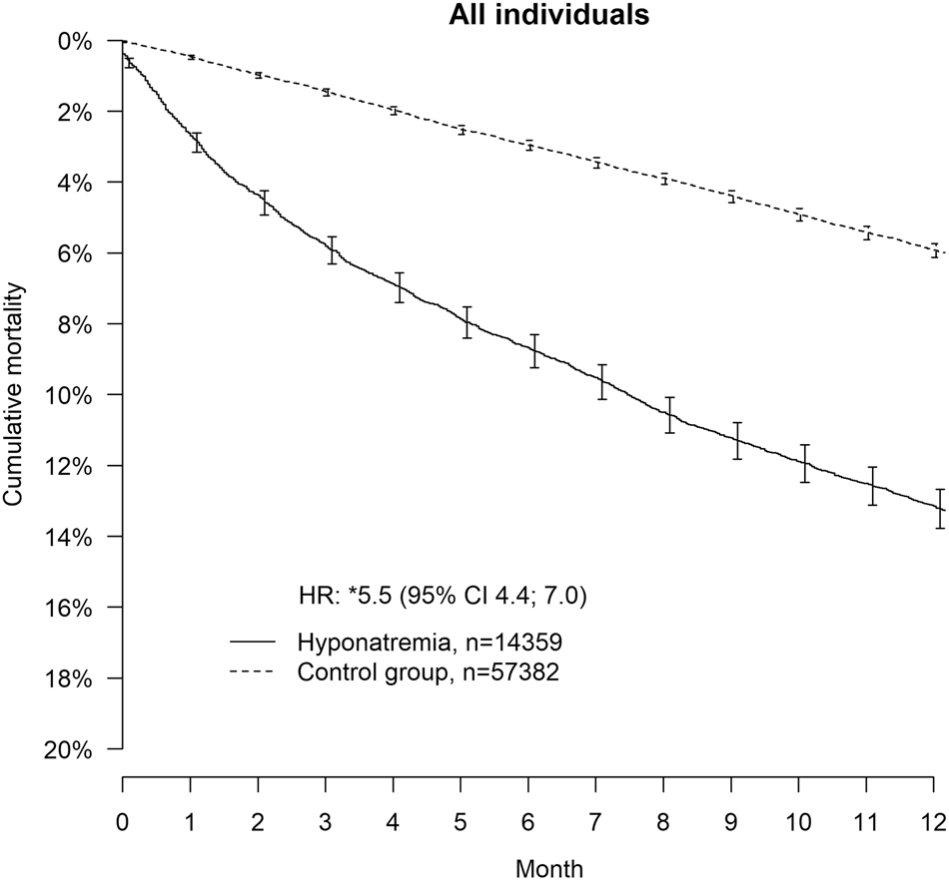
Pooled unadjusted Kaplan–Meier curves and adjusted hazard ratios comparing individuals hospitalized for hyponatremia with controls for all-cause mortality over 12 months. Hazard ratio HR. The asterisk symbol indicates that adjustment were made for age, renal disease, cardiovascular disease, liver disease, adrenal failure, congestive obstructive pulmonary disease, pneumonia, diabetes, alcohol abuse, frailty, cancer and treatment with thiazide diuretics, ACE/ARB, SSRIs, antiepileptic drugs, beta blockers, antiarrhythmic agents, and anticoagulants

**Fig. 2:**
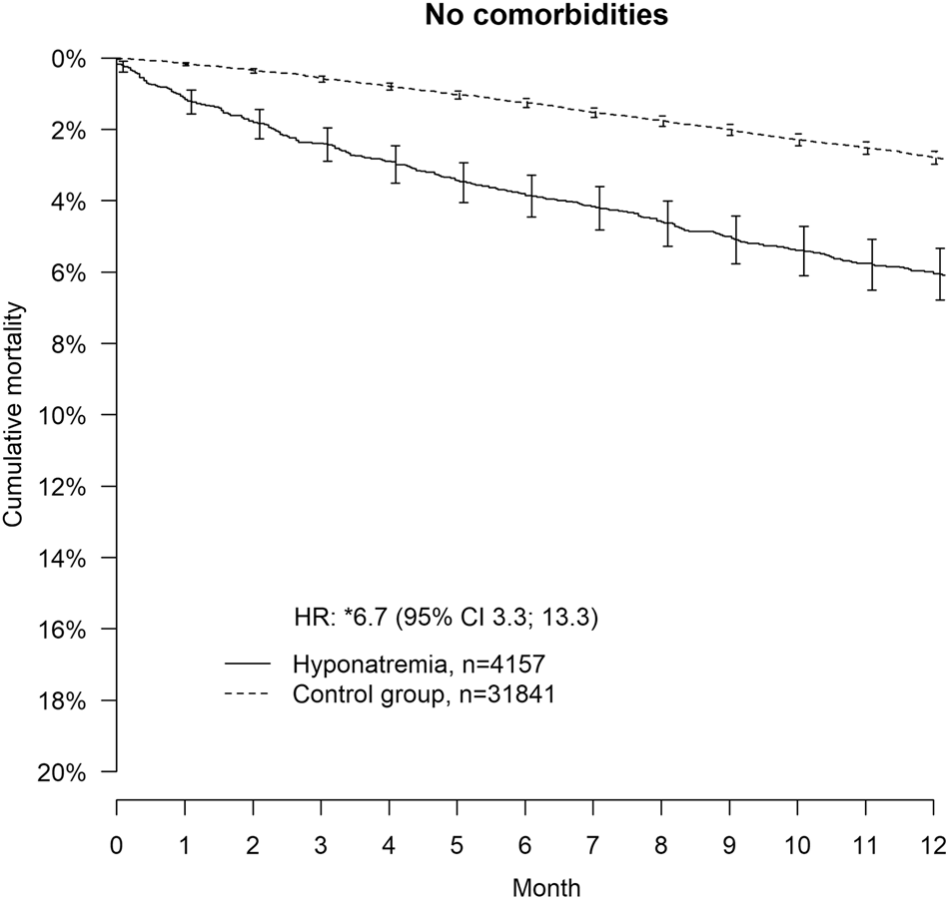
Pooled, unadjusted Kaplan–Meier curves and adjusted hazard ratios comparing individuals hospitalized for hyponatremia without previously detected comorbidity with controls for all-cause mortality over 12 months. Hazard ratio HR. The asterisk symbol indicates that adjustment were made for age, frailty, and treatment with thiazide diuretics, ACE/ARB, SSRIs, antiepileptic drugs, beta blockers, antiarrhythmic agents, and anticoagulants

**Fig. 3:**
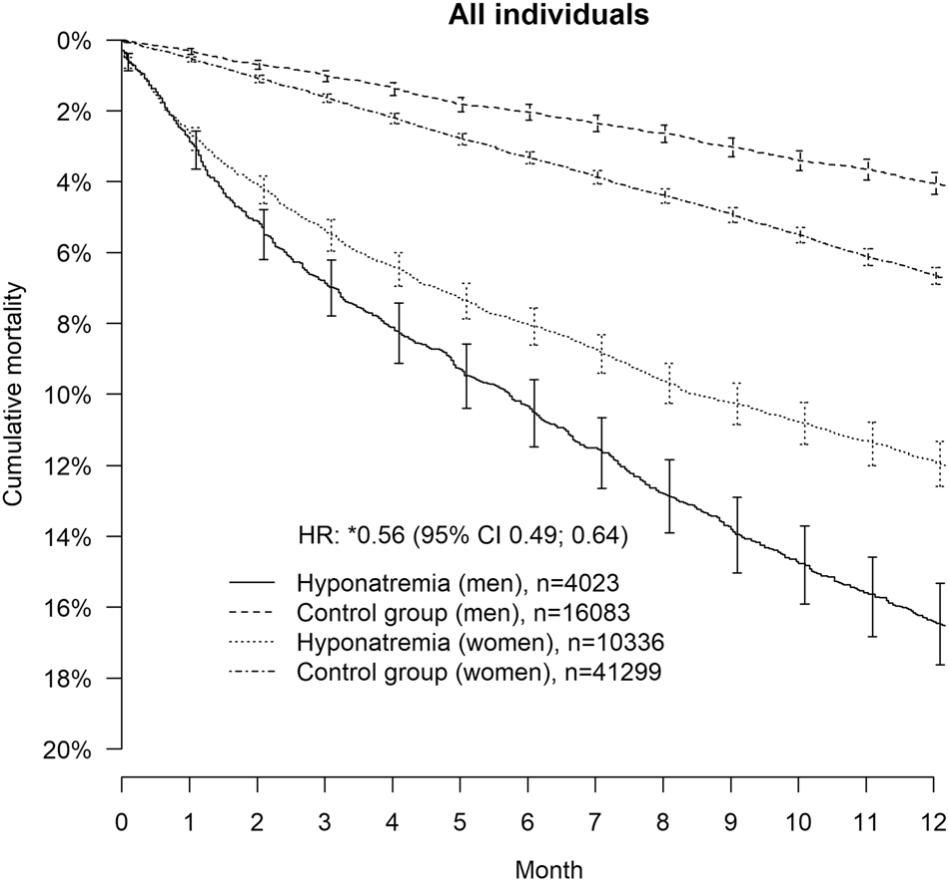
Pooled, unadjusted Kaplan–Meier curves and adjusted hazard ratios comparing men and women hospitalized for hyponatremia and controls for all-cause mortality over 12 months. Hazard ratio HR. The asterisk symbol indicates HR for women vs. men (reference); Adjustment were made for age, renal disease, cardiovascular disease, liver disease, adrenal failure, congestive obstructive pulmonary disease, pneumonia, diabetes, alcohol, frailty, cancer and treatment with thiazide diuretics, ACE/ARB, SSRIs, antiepileptic drugs, beta blockers, antiarrhythmic agents and anticoagulants

**Fig. 4:**
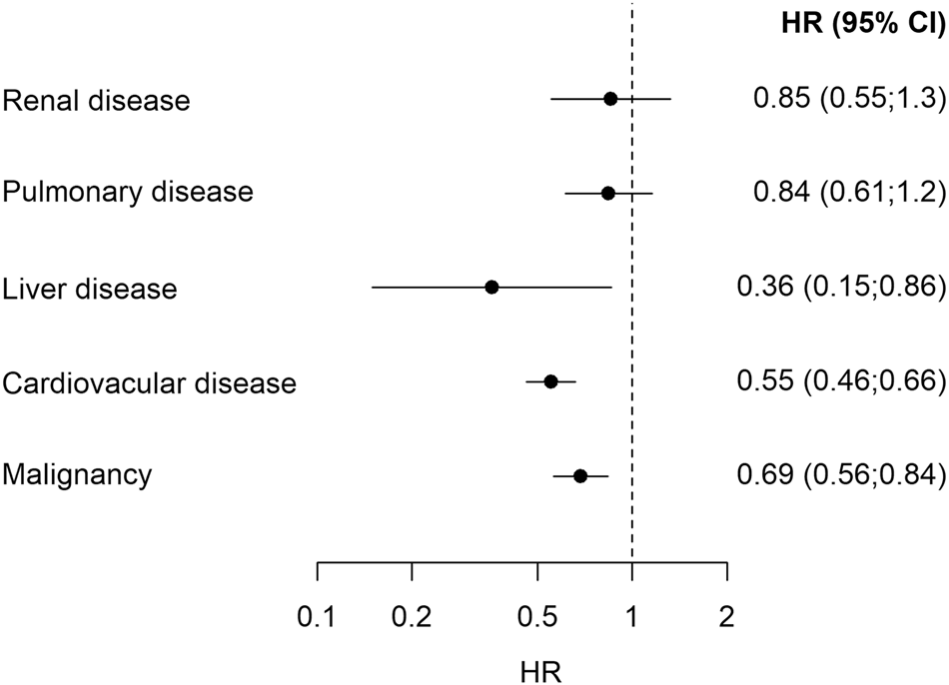
Forest plot for risk of death for women vs. men with hospital requiring hyponatremia according to comorbidity. HR Hazard ratio; HR < 1.0 favors women

During the 9-year study period, 14,359 individuals with a principal diagnosis of hyponatremia, 18 years or older, and 57,382 matched controls were identified (study flow chart, Fig. 1). Demographics, comorbidities and treatment are presented in Table 1. The median age was 76 years (IQR: 65–84) and the majority were women (72%). Median age for women and men was 79 and 68 years, respectively. Female subjects hospitalized for hyponatremia were more often exposed to thiazide diuretics and selective serotonin reuptake inhibitors (Table 1), while men with hyponatremia had a higher prevalence for comorbidities, such as ischemic stroke, diabetes, and alcoholism. The unadjusted 1-year lethality was 6.0% for the controls and 13.3% for the patients hospitalized for hyponatremia. Male cases faced a 1-year lethality of 16.5%, compared with 12.0% in female cases. The adjusted hazard ratios (HRs) for 1-year lethality showed that patients hospitalized for hyponatremia were at higher risk compared with controls with HRs of 5.5 (95% CI: 4.4–7.0) (Fig. 1). In the healthier subpopulation with hyponatremia and without previously recorded comorbidity the adjusted HR for 1-year lethality was 6.7 (95% CI: 3.3–13.3) (Fig. 2). Similar patterns were seen in both men and women, but the impact of hyponatremia was significantly smaller in women compared with men; HR for the interaction hyponatremia-female gender was 0.56 (95% CI: 0.49–0.64) (Fig. 3). In the healthier subgroup the lethality remained lower for women with a HR of 0.49 (95% CI: 0.34–0.71). Other subgroup analyses showed that hyponatremia was associated with a significantly increased lethality in patients with concomitant cardiovascular disease, liver disease, pulmonary disease, renal disease, or alcoholism; adjusted HR: 5.0 (95% CI: 3.6–6.9), 10.6 (95% CI: 2.2–51.2), 2.7 (95% CI: 1.5–4.7), 2.4 (95% CI: 1.2–5.0), and 4.1 (95% CI: 1.7–9.9), respectively. The forest plot (Fig. 4) illustrate that hospitalization for hyponatremia had a significantly larger impact on lethality in men compared with women in the subgroups with malignancy, cardiovascular disease, and liver disease.

**Table 1:** Medical characteristics and treatment among patients with and without exposition for hyponatremia

	All controls (not HHN)	All cases (HHN)	Men (controls) not HHN	Women (controls) not HHN	Men (cases) HHN	Women (cases) HHN
No of patients	57,382 (100)	14,359 (100)	16,083 (100)	41,299 (100)	4023 (100)	10,336 (100)
Demographics						
Age (years)	76 (65–84)	76 (65–84)	68 (59–78)	79 (68–86)	68 (59–78)	79 (68–85)
Female	41,299 (72.0)	10,336 (72.0)	0	41,299 (100)	0	10,336 (100)
Ischemic heart disease	7,880 (13.7)	2,808 (19.6)	2471 (15.4)	5,409 (13.1)	836 (20.8)	1,972 (19.1)
Stroke	4,540 (7.9)	1,884 (13.1)	1,117 (6.9)	3,423 (8.3)	633 (15.7)	1251(12.1)
Congestive heart failure	4,493 (8.0)	1,900 (13.2)	1,063 (6.6)	3,430 (8.3)	608 (15.1)	1,292 (12.5)
Kidney disease						
Diabetes	6,581 (11.4)	2,423 (16.9)	1,997 (12.4)	4,584 (11.1)	842 (20.9)	1,581 (15.3)
Cancer	11,251 (19.6)	3,826 (26.6)	2,901 (18.0)	8,350 (20.2)	1,123 (27.9)	2703 (26.1)
Pulmonary disease	2,740 (4.8)	1,692 (11.8)	643 (4.0)	2,097 (5.1)	520 (12.9)	1,172 (11.3)
Alcoholism	1,028 (11.8)	2,285 (15.9)	619 (3.8)	409 (1.0)	1367 (34.0)	918 (8.9)
Treatment						
Anticoagulants	15,437 (26.9)	5,074 (35.3)	4,070 (25.3)	11,367 (27.5)	1,335 (33.2)	3,739 (36.2)
Thiazide diuretics	7,425 (12.9)	5,204 (36.2)	1,531 (9.5)	5,894 (14.3)	923 (22.9)	4,281 (41.4)
Beta blockers	14,045 (24.5)	5,282 (36.8)	3,391 (21.1)	10,654 (25.8)	1,311 (32.6)	3,971 (38.4)
ACE/ARB	13,251 (23.1)	5,712 (39.8)	3,754 (23.3)	9,497 (23.0)	1,472 (36.6)	4,240 (41.0)
Antiarrhythmics	1,558 (2.7)	473 (3.3)	319 (2.0)	1,239 (3.0)	134 (3.3)	339 (3.3)
Insulin treatment						
OGD						
SSRI	4,861 (8.5)	2,439 (17.0)	687 (4.3)	4174 (10.1)	535 (13.3)	1904 (18.4)
Antiepileptic drugs	985 (1.7)	1126 (7.8)	271 (1.7)	714 (1.7)	440 (10.9)	686 (6.6)

Data are *N* (%) or median (interquartile range). Pulmonary disease includes chronic obstructive pulmonary disease, pulmonary embolism, and primary pulmonary hypertension

*HHN* hospitalized hyponatremia, *ACE* angiotensin converting enzyme inhibitors, *ARB* angiotensin II receptor blockers, *OGD* oral glucose lowering drugs

## Discussion

This nationwide observational registry study reports two important findings: an almost fivefold increased lethality within 1-year after hospitalization for hyponatremia and a gender-associated difference where hyponatremia increased lethality nearly twice as much in men compared with women.

The present study is unique in focusing on lethality in patients hospitalized due to hyponatremia. After adjustment for a factors of prognostic importance, hospitalization due to hyponatremia was independently associated with a markedly increased lethality. Furthermore, we studied a subgroup free from previously identified comorbidities. Interestingly, the independent association between hyponatremia and lethality remained in this healthier group.

The association between hyponatremia and lethality has been explored extensively over the last decades, and reports invariably show that hyponatremia predicts both in-hospital [13–16] and future lethality [17–19], for a wide range of diseases.

The question whether hyponatremia directly contributes to lethality or merely reflects the severity of an underlying disease is under debate [10]. Previous epidemiologic studies have demonstrated cumulative lethality rates from 6 to 29% depending on the underlying disease [10, 13, 20]. In a narrative review of hyponatremia and lethality Hoorn and Zietse [10] found nine studies that appeared to favor a direct contribution of hyponatremia to lethality while six studies instead pointed towards an effect mediated through the underlying disease [10, 13, 14, 16, 18, 20–30]. The authors concluded that there were no convincing data confirming that hyponatremia per se attributes to lethality [10]. Which may then be the potential mechanisms that links hyponatremia with death? One suggested mechanism is hyponatremia-induced oxidative stress [31]. Another hypothesis is the activation of the vasopressor system. A prospective cohort study by Eckart et al. published in 2018, with laboratory data from 6962 individuals at the emergency department reported a strong association between vasopressin levels, mirrored by copeptin, and lethality [32]. In an experimental model on aged rats Barsony et al. showed that chronic hyponatremia is associated with reduction in bone mineral density, hypogonadism, sarcopenia, and cardiac fibrosis [33]. Hence, there is no specific culprit causative mechanism that can explain the association between hyponatremia and death.

So far very few studies have reported on the impact of gender on hyponatremia and lethality [8, 16]. We found that men hospitalized due to hyponatremia faced a subsequent twofold risk for death compared with women. A similar increased lethality in men with hyponatremia was reported in the previously mentioned study by Eckart et al. [33]. Gender differences in sodium metabolism are perhaps most obvious in acute hyponatremia, with women at considerable risk of cerebral edema, whereas men rarely suffer this complication [34]. However, acute hyponatremia is rare, and differences at a population level between men and women more likely relates to chronic hyponatremia. Wald et al. [16] examined in-hospital lethality among 53,236 adults at a medical center in Boston, USA, and Mohan et al. [8] utilized data from a population-based cross-sectional study of 14,697 adults from the US to examine all-cause lethality over a period of 7 years. Contrary to our findings, both groups found hyponatremic women to be at significantly greater risk of dying than hyponatremic men. However, differences in methodology, and severity of hyponatremia, make comparisons hard. In the study by Wald et al. a majority (80%) of patients suffered from mild hyponatremia (133–137 mmol/L), and in the study by Mohan et al. hyponatremia was defined as a s-sodium of <133 mmol/L or <136 mmol/L, depending on current reference ranges. In our cohort, sodium was significantly lower, with an estimated mean of 122 mmol/L, based on validation data [9, 12]. In the hospital setting, drug induced hyponatremia appears to be more common, and sodium levels lower, in women than in men [35, 36]. In our study, it was evident that females were more frequently exposed to treatment with SSRIs and thiazide diuretics, while males had a greater burden of comorbidities associated with a worse prognosis. However, the adjustment procedure ought to control for most of these effects. In addition, the subgroup without previously known comorbidity showed similar gender-associated differences. Thus, the higher lethality in men hospitalized due to hyponatremia cannot be fully explained by a greater burden of comorbidity. A potential explanation could be that male cases in our study were exposed to lower sodium concentrations. Unfortunately, we did not have access to laboratory data and could not adjust for sodium levels.

Some limitations should be considered when interpreting our results. Firstly, the lack of laboratory and anthropometric data disables us from analyzing the effect of different sodium levels and anthropometric data on prognosis. Secondly, the study is based on a previous matched case-control study, which may have inflated the relative risk for the cases compared with prospective cohort studies where both cases and controls are sampled from a hospitalized population. However, in our multivariable analyses we adjusted for frailty.

In conclusion, patients hospitalized for hyponatremia faced a markedly increased subsequent lethality that was independent of concomitant disease. This increase was nearly twice as large among men compared with women.

## Supplementary information

Appendix
