# Supplementary material for: Sex-specific risks of death in patients hospitalized for hyponatremia: a population-based study
Source: Endocrine. 2019 Sep 2;66(3):660–5. doi: 10.1007/s12020-019-02073-x (PMC6887836; doi:10.1007/s12020-019-02073-x)
Supplement: Supplementary file 1 — Appendix [file 12020_2019_2073_MOESM1_ESM.docx]

**Appendix 1.** Flow chart of the study

**Appendix 2.** Definitions of variables included in the multivariable analyses.

| Variables | Codes |
| --- | --- |
| Diseases |  |
| Renal diseases | N17-19, procedure codes DR016, DR024, KAS00, KAS10, KAS20 |
| Sepsis | A41 |
| Pneumonia | J18 |
| Meningitis | G00-G07 |
| Ischemic heart disease | I20-25 |
| Malignant disease | C |
| Congestive heart failure | I50 |
| Liver diseases | K70-77 Procedure codes JJB, JJC |
| Cerebrovascular diseases | I60-64, I69 |
| Pulmonary disease:  Chronic obstructive pulmonary disease  Pulmonary embolism  Chronic Obstructive Pulmonary Disease (COPD)  Primary Pulmonary Hypertension | J44  I26  J44  I27 |
|  | Combination of ATC- and ICD-10 codes, each beginning with |
| Alcoholism | ATC: N07BB03, N07BB04, N07BB01, N07BB05, N07BB  ICD10: E244, F10, G312, G621, G721, I426, K292, K70, K860, O354, P043, Q860, T51, Y90-91, Z502, Z714 |
| Adrenal insufficiency | ICD10: E27 |
| Diabetes | ICD10: E10-E14 |
| Other factors |  |
| Drug use | Number of dispensed drugs 90 days prior to index date, categorised into <4, 4-7, 8-12 and >12 drugs |
| Previous hospitalization, (frailty | >2 days within 2 years prior to index date |
